# Unraveling T Cell Responses for Long Term Protection of SARS-CoV-2 Infection

**DOI:** 10.3389/fgene.2022.871164

**Published:** 2022-05-04

**Authors:** Dongyuan Wu, Runzhi Zhang, Susmita Datta

**Affiliations:** Department of Biostatistics, University of Florida, Gainesville, FL, United States

**Keywords:** COVID-19, RNA-seq, T cell, differential expression, gene co-expression network, functional annotation, deep learning

## Abstract

Due to the COVID-19 pandemic, the global need for vaccines to prevent the disease is imperative. To date, several manufacturers have made efforts to develop vaccines against SARS-CoV-2. In spite of the success of developing many useful vaccines so far, it will be helpful for future vaccine designs, targetting long-term disease protection. For this, we need to know more details of the mechanism of T cell responses to SARS-CoV-2. In this study, we first detected pairwise differentially expressed genes among the healthy, mild, and severe COVID-19 groups of patients based on the expression of CD4^+^ T cells and CD8^+^ T cells, respectively. The CD4^+^ T cells dataset contains 6 mild COVID-19 patients, 8 severe COVID-19 patients, and 6 healthy donors, while the CD8^+^ T cells dataset has 15 mild COVID-19 patients, 22 severe COVID-19 patients, and 4 healthy donors. Furthermore, we utilized the deep learning algorithm to investigate the potential of differentially expressed genes in distinguishing different disease states. Finally, we built co-expression networks among those genes separately. For CD4^+^ T cells, we identified 6 modules for the healthy network, 4 modules for the mild network, and 1 module for the severe network; for CD8^+^ T cells, we detected 6 modules for the healthy network, 4 modules for the mild network, and 3 modules for the severe network. We also obtained hub genes for each module and evaluated the differential connectivity of each gene between pairs of networks constructed on different disease states. Summarizing the results, we find that the following genes *TNF*, *CCL4*, *XCL1*, and *IFITM1* can be highly identified with SARS-CoV-2. It is interesting to see that *IFITM1* has already been known to inhibit multiple infections with other enveloped viruses, including coronavirus. In addition, our networks show some specific patterns of connectivity among genes and some meaningful clusters related to COVID-19. The results might improve the insight of gene expression mechanisms associated with both CD4^+^ and CD8^+^ T cells, expand our understanding of COVID-19 and help develop vaccines with long-term protection.

## 1 Introduction

As of April 3^rd^, 2022, the coronavirus disease 2019 (COVID-19) global pandemic has lasted for about 2 years, with more than 491 million confirmed cases, including around 6.15 million deaths worldwide (Dong et al., 2020). This disease is caused by the severe acute respiratory syndrome coronavirus 2 (SARS-CoV-2), whose genome sequence identity is more similar to two bat-derived coronavirus strains than any known human-infecting virus (Lu et al., 2020). Vaccination can offer the most effective way with a long-term strategy to prevent and control the spread of COVID-19 (Hu et al., 2021). Towards this effort, several manufacturers have proposed different vaccine designs against SARS-CoV-2 (Creech et al., 2021). Specifically, the novel mRNA-based vaccines provide significant efficacy and stability for some time (Le et al., 2020; Pardi et al., 2020). The spike protein is one of the essential structural proteins of the SARS-CoV-2 virus, which includes two subunits to regulate receptor binding and membrane fusion, respectively (Huang et al., 2020). SARS-CoV-2 enters host cells through receptor angiotensin-converting enzyme 2 (ACE2) using its two spike protein subunits (Wheeler et al., 2021). Because of that, improving antibody responses to the spike protein becomes the primary research direction of current vaccine development, and researchers put less effort into following up with the T cell immunity (Saini et al., 2021). However, Diao et al. (2020) found that COVID-19 patients, especially patients who needed Intensive Care Unit (ICU) care, had significantly fewer CD4^+^ and CD8^+^ T cells than healthy people. Thus, understanding the mechanism of T cell responses to SARS-CoV-2 can provide additional knowledge on vaccine design (Grifoni et al., 2020).

Different expressions of the cell-surface receptors CD4 and CD8 indicate that T cells have different types, including CD4^+^ T cells and CD8^+^ T cells (Jarjour et al., 2021). CD4^+^ T cells play a crucial “helper” role in regulating anti-viral immune responses through the secretion of specific cytokines, whereas CD8^+^ T cells are “killers” that aim to directly attack and kill the pathogen-infected cells (Luckheeram et al., 2012; Tay et al., 2020; Jarjour et al., 2021). In this study, we analyzed the single-cell RNA sequencing (scRNA-seq) data of both CD4^+^ and CD8^+^ T cells through some downstream analyses, including differential expression analysis of the genes, network analysis to detect gene co-expression networks, and deep learning to classify the samples with different disease states. In addition to T cells, B cells are also engaged in the battle with the SARS-CoV-2 virus by mediating partial immunological memory (Quast and Tarlinton, 2021). Several studies have shown the appearance of B cells after the onset of symptoms of the mild COVID-19 (Hoehn et al., 2021; Rodda et al., 2021). Moreover, the longitudinal study of the bulk level RNA sequencing (RNA-seq) data is also valuable to discover the evolution of the disease. Recent studies found that the COVID-19 patients gradually developed symptoms and needed several weeks or months to recover after the infection (Nehme et al., 2021; Petersen et al., 2021). Thus, we included two additional datasets, i.e., longitudinal blood samples using bulk level RNA-seq data and B cells at the single-cell level, to enrich our study. The overall goal of our study is to enhance the insight of gene expression associated with CD4^+^ and CD8^+^ T cells along with B cells, and provide some potential genes associated with the immune responses of the patients at different stages of SARS-CoV-2.

The CAMDA challenge provided the CD4^+^ T cells data (Bacher et al., 2020) and the longitudinal bulk data (McClain et al., 2021); we integrated them with the additional CD8^+^ T cells data (Kusnadi et al., 2021) and the B cells data (Hoehn et al., 2021).

## 2 Materials and Methods

**FIGURE 1:**
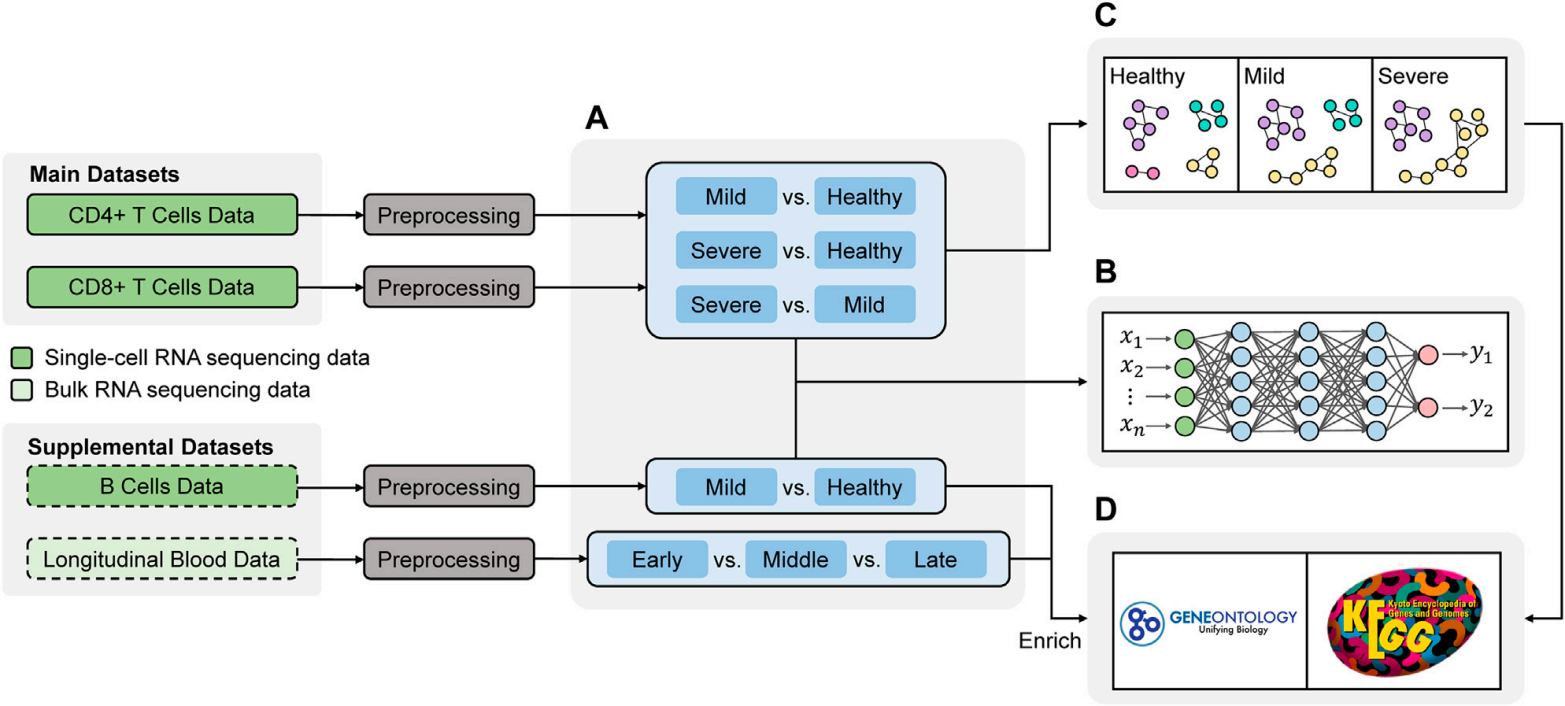
Workflow of the study. The analysis primarily focused on two main datasets, and two supplemental datasets were included to enrich the findings **(A)** Differential Expression analysis to obtain the differentially expressed (DE) genes among different disease states **(B)** Deep learning for validating the DE genes **(C)** Network analysis comparing the connectivity of genes in different disease states **(D)** Functional annotation.

We show the workflow of this project in Figure 1. Most of analytical steps were conducted using R (R Core Team, 2021). The network development in Figure 1B was implemented using Julia (Bezanson et al., 2017). The codes for the analyses are available on https://github.com/dongyuanwu/UTRCOV2. We will introduce all the steps in the following sections.

### 2.1 Data and Preprocessing

The primary datasets we used are the scRNA-seq data of CD4^+^ T cells (Bacher et al., 2020) and CD8^+^ T cells (Kusnadi et al., 2021) from blood samples. Both studies categorize the COVID-19 patients into two groups: mild (non-hospitalized) and severe (hospitalized). The CD4^+^ T cells dataset contains 104,417 cells, including 23,573 cells from 6 mild COVID-19 patients, 76,887 cells from 8 severe COVID-19 patients, and 3,957 cells from 6 healthy donors; while the CD8^+^ T cells dataset has 72,905 cells including 13,108 cells from 15 mild COVID-19 patients, 55,169 cells from 22 severe COVID-19 patients, and 4,628 cells from 4 healthy donors.

Since the datasets are pretty sparse, we filtered out genes not expressed in most cells. We implemented different criteria since different datasets have different degrees of sparsity. For the CD4^+^ T cells, genes not expressed in at least 1% of the total cells were removed, leaving a total of 8,959 genes (23,945 genes before filtering) in the analysis; for the CD8^+^ T cells, genes not expressed in at least 2.5% of the total cells were filtered out, leaving a total of 9,112 genes (13,816 genes before filtering) in the analysis. We used the R package Seurat V3 (Stuart et al., 2019) to normalize and scale the raw expression counts.

Furthermore, in order to enrich the discoveries from T cells, we used another bulk RNA-seq data of whole blood samples (McClain et al., 2021), which contains the information of time from COVID-19 symptom onset (early ≤ 10 days, middle 11–21 days, late > 21 days), for tracing the changes of gene expressions longitudinally. On another note, in addition to the T cells, B cells also play a critical role in the immune system. Thus, we also analyzed scRNA-seq data of B cells (Hoehn et al., 2021), including healthy donors and mild (non-hospitalized) COVID-19 patients.

### 2.2 Differential Expression Analysis

We adopt the pairwise differential expression analysis among the mild COVID-19 patients, the severe COVID-19 patients, and the healthy donors. Due to the highly unbalanced distribution of the scRNA-seq data, we used the random under-sampling algorithm to make sure the number of cells in the majority class is almost equal to the minority class in each pairwise comparison. We repeated the random selections 100 times to eliminate the potential bias due to unbalanced data. In each iteration, we implemented the differential expression analysis between two groups using MAST (Finak et al., 2015), a two-part hurdle model. The first part of this model is a logistic regression model to account for the gene expression rate, and the second part is a linear model with the normal distribution assumption for the expression level, conditioning on a cell expressing the gene. We declared a gene differentially expressed (DE) if it was selected as DE gene in all the 100 rounds with the $\left|log_{2}FC\right|>0.25$ and the false discovery rate (FDR) adjusted p -value ( q -value) <0.05 , where FC is the fold change that measures the amount of changes of the expression value of a gene between two groups.

### 2.3 Network Analysis

As the genes rarely work independently, we constructed co-expression networks among the genes. For a lesser computational burden, we included only DE genes to build the network. A few novel gene co-expression network construction approaches designed explicitly for scRNA-seq data have been proposed in recent years, but most of them require pseudo-time information for the cell differentiation process. To avoid the uncertainty of its estimation, we applied the partial information decomposition and context (PIDC) algorithm (Chan et al., 2017) to study the gene-gene associations among the DE genes. And then, we built gene co-expression networks based on partial information decomposition (PID) for the healthy donors, the mild COVID-19 patients, and the severe COVID-19 patients, respectively. PIDC calculates a proportional unique contribution (PUC) score $u_{i,j}$ for each pair of genes i and j , and the edge weight $W_{i,j}$ between a pair of genes i and j can be given by

$$W_{i,j}=F_{i}\left(u_{i,j}\right)+F_{j}\left(u_{i,j}\right),$$

where $F_{i}\left(U\right)$ represents the cumulative distribution (Gamma or Gaussian empirical probability distribution) function of all the PUC scores related to gene i . In this way, the edge weights are all bounded between 0 and 2.

To detect the modules for each network (Gill et al., 2010), we first divided the edge weights by 2 to let them range in [0, 1] . And then set the minimum module size and the minimum accepted edge weight depending on different datasets. We defined the connectivity of a gene by the number of genes it is connected to. In each module, we counted the connectivity for each gene. In addition, we identified the hub gene in a specific module using the following criteria:

1) Hub genes are the genes that have the highest connectivity, or the genes whose connectivities are very close to the highest connectivity, i.e., $C_{hub}\geq 0.9\times max\left(C_{m}\right)$ , where $C_{hub}$ represents the connectivities of the hub genes, and $C_{m}$ is a vector of the connectivities of genes in module m ;
2) The number of hub genes should not exceed 30% of the total number of genes in one module.

Moreover, we used a permutation test (Gill et al., 2010) with 500 random permutations to evaluate the differential connectivity of a single gene between two networks. The difference in the connectivity of gene i between two networks $\pi_{1}$ and $\pi_{2}$ can be assessed by

$$d\left(i\right)=\frac{1}{G-1}\underset{j\neq i}{\sum }\left|W_{i,j}^{\pi_{1}}-W_{i,j}^{\pi_{2}}\right|,$$

where G is the total number of genes. Genes with the FDR adjusted p -values less than 0.05 were treated as differentially connected genes between two networks.

### 2.4 Deep Learning

To detect whether the DE genes are also able to classify the patient samples into different disease states from the pairwise comparison, we implemented a classification algorithm, i.e., Multi-layer Perceptron (MLP). Firstly, in each dataset, we treated the cells from each patient in turn as the test set and those from other patients as the training set. In other words, the number of independent training sets (or their corresponding test sets) is identical to the number of patients. By dividing the dataset in this way, we aimed to investigate if the cell disease states of one patient could be predicted by cells from the remaining patients. Secondly, we implemented 5-folds cross-validation on each training set for parameter tuning. In this way, we can optimize the MLP models and use them to predict the corresponding test sets. Specifically, we fitted MLP models with the combinations of two optimizers, including adaptive moment estimation (Adam) (Kingma and Ba, 2017) and root mean square propagation (Rmsprop) (Tieleman and Hinton, 2012), and different group weights, i.e., using weight and not using weight. In addition, we used a mixture of the rectified linear unit (ReLU) and sigmoid activations, which are commonly used in a neural network for binary classification, along with dropout (Hinton et al., 2012) in MLP. Lastly, the MLP model with the highest accuracy in CV was used for the test set prediction.

## 3 Results

### 3.1 Differentially Expressed Gene

**FIGURE 2:**
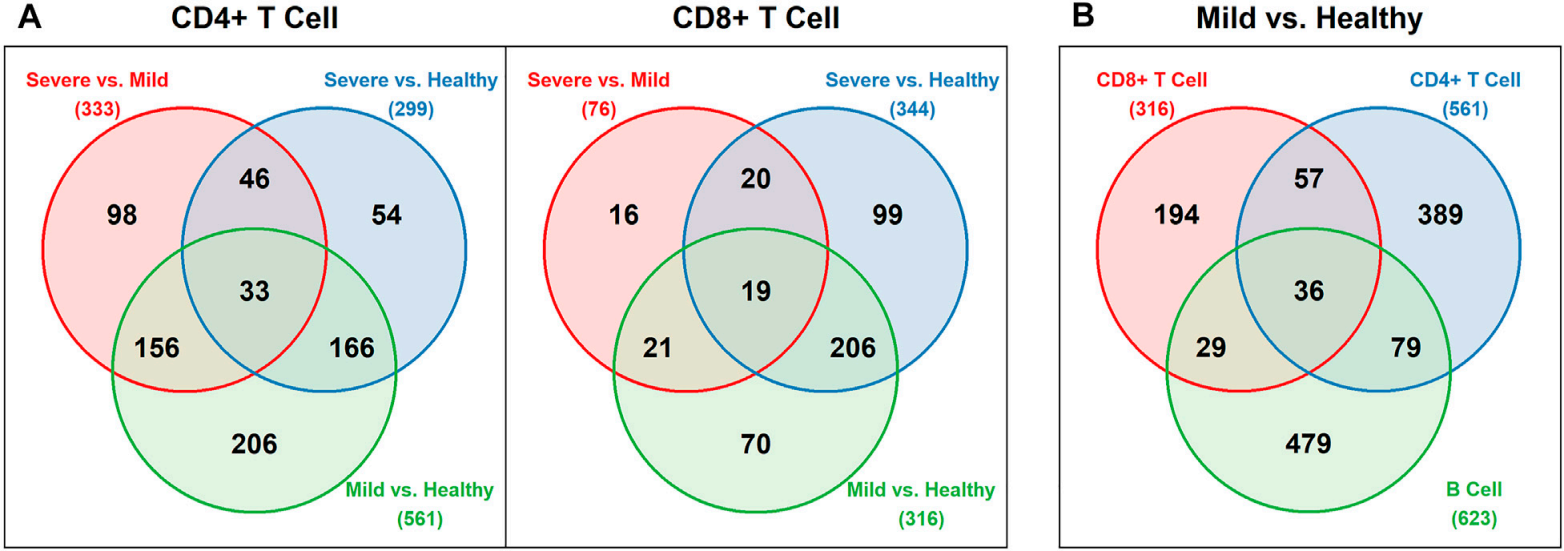
Venn diagrams for DE genes **(A)** Pairwise comparisons of the healthy, mild, and severe groups for CD4^+^ and CD8^+^ T cells **(B)** Comparison of the mild vs healthy group for CD4^+^ T cells, CD8^+^ T cells, and B cells.

After the differential expression analysis, we obtained different numbers of DE genes for each pairwise comparison among the healthy, mild and severe groups, based on CD4^+^ T cells and CD8^+^ T cells, separately. We also conducted the differential expression analysis for B cells with only healthy and mild groups included. Figure 2 displays the Venn diagrams of these comparisons. As we can see, there are more DE genes detected in the mild vs healthy comparison than the severe vs mild comparison. It is worth noting that the number of DE genes between the mild and the severe groups from CD8^+^ T cells is much lower than any other comparisons. Additionally, in the comparison of the mild and the healthy groups, although the number of DE genes from CD8^+^ T cells is lower than both CD4^+^ T cells and B cells, there are still 36 shared DE genes from all 3 cell types. The detailed DE genes list can be found in the Supplementary Material. Interestingly, the 33 shared DE genes in CD4^+^ T cells comparisons and the 19 shared DE genes in CD8^+^ T cells comparisons do not overlap. It could be due to the various functions and corresponding encoding genes CD4^+^ and CD8^+^ T cells express.

**FIGURE 3:**
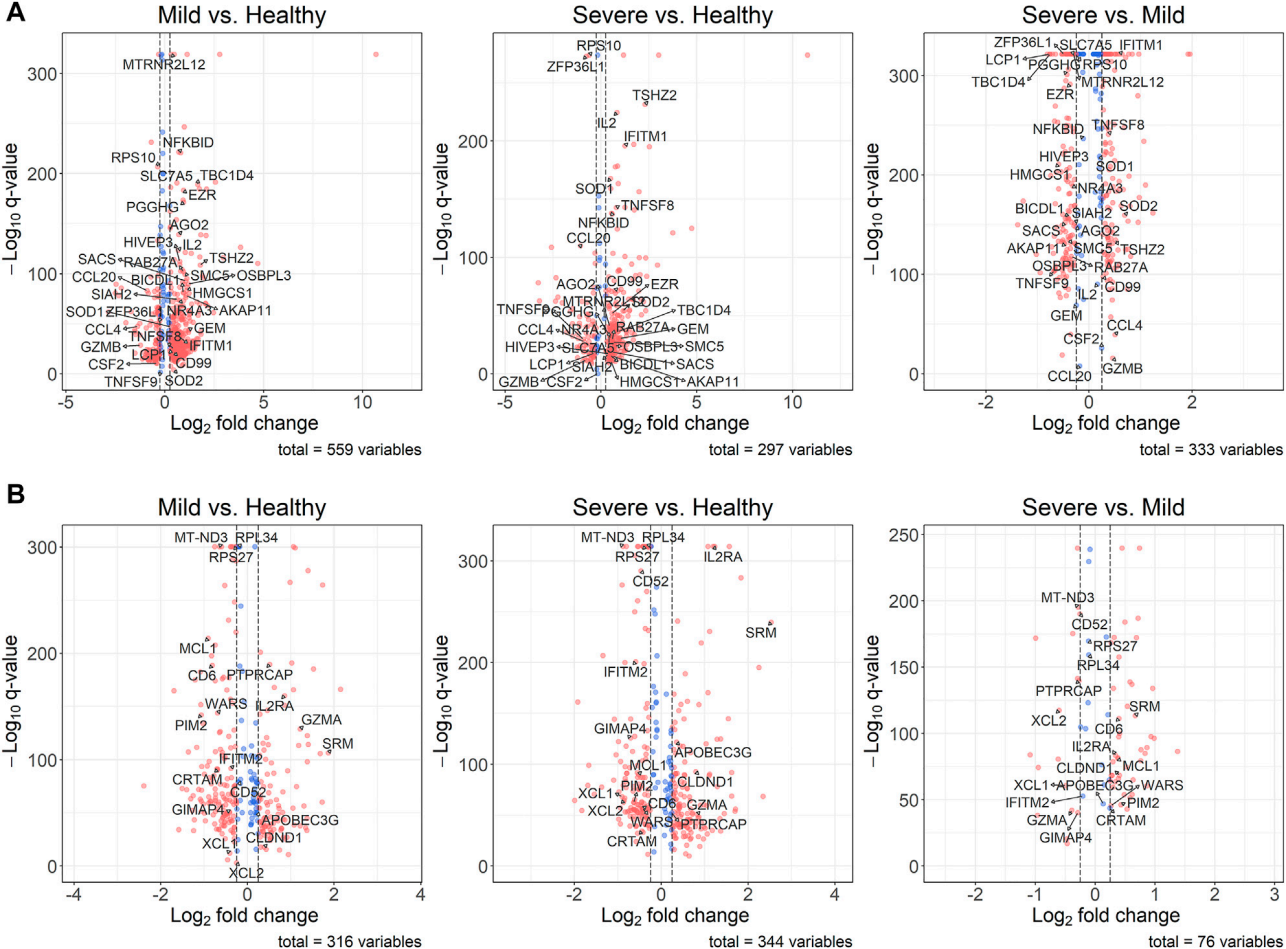
Volcano plots for DE genes. The thresholds of the absolute $log_{2}FC$ is 0.25 **(A)** Comparisons based on CD4^+^ T cells. DE genes *DDX3Y* and *EIF1AY* have been removed from the mild vs healthy plot and the severe vs healthy plot because of their infinite $log_{2}FC$ **(B)** Comparisons based on CD8^+^ T cells.

Although, we detected DE genes using the under-sampling method, it is still important to know the original distributions of the expression levels of these genes. Since the differential expression analysis was based on the 100 balanced datasets, we used the average q -values across 100 analyses. However, we used the original fold changes of the DE genes to draw the volcano plots. In Figure 3, we annotated the shared DE genes among three comparisons. Although the original fold changes of some DE genes do not reach the threshold we set ( $\left|log_{2}FC\right|>0.25$ ), most of the shared DE genes have met this criterion, especially for the comparisons between COVID-19 patients and healthy donors. Moreover, most of the shared DE genes of mild vs healthy across CD4^+^ T cells, CD8^+^ T cells, and B cells display significant differences based on the original unbalanced data (Supplementary Figure S1).

### 3.2 Classification Based on DE Genes

Based on the selected MLP models, we implemented the prediction to see if the DE genes could help to identify the different COVID-19 disease states. The details of the selected MLP models for each comparison can be found in Supplementary Table S1. Since each test set contains just one actual class, we only calculated the accuracy for each test set (Supplementary Tables S2–S8). However, we stacked the predicted results from all test sets to obtain the overall accuracy, sensitivity, specificity, and area under the curve (AUC) of the receiver operating characteristic (ROC). According to Table 1, the corresponding DE genes can distinguish the COVID-19 patients (mild or severe) from healthy donors using CD4^+^ T cells, CD8^+^ T cells, or B cells. However, it is difficult to classify the mild and severe COVID-19 patients based on CD4^+^ T cells and CD8^+^ T cells. On the other hand, we found a worse prediction in CD8^+^ T cells than in CD4^+^ T cells for the comparisons between the severe and mild groups. It could be due to the fewer DE genes detected in this comparison of CD8^+^ T cells. In summary, MLP models can efficiently distinguish the healthy people and the COVID-19 (mild or severe) patients using their corresponding DE genes.

**TABLE 1:** Results of the prediction of the selected MLP models. H, M, and S indicate the healthy, the mild and the severe COVID-19 groups, respectively.

Cell Type	Comparison	Stacked accuracy	Stacked sensitivity	Stacked specificity	Stacked AUC
CD4^+^ T cells	M vs. H	0.9066	0.9087	0.8944	0.9015
	S vs. H	0.9783	0.9870	0.8100	0.8985
	S vs. M	0.8070	0.8397	0.7003	0.7700
CD8^+^ T cells	M vs. H	0.9827	0.9904	0.9611	0.9757
	S vs. H	0.9757	0.9866	0.8468	0.9167
	S vs. M	0.7595	0.8521	0.3701	0.6111
B cells	M vs. H	0.9775	0.9857	0.9694	0.9775

### 3.3 Gene Co-Expression Network

We summarized unions of DE genes from different comparisons of CD4^+^ T cells (759 genes) and CD8^+^ T cells (451 genes), respectively. Based on these DE genes, we built a network for each group. We set the minimum module size of the network as 5 for both cell types. Due to various number of DE genes included in networks of different cell types, we set different minimum accepted edge weights (0.999 for CD4^+^ T cells and 0.997 for CD8^+^ T cells). These settings result in 130 and 156 DE genes included in the CD4^+^ and CD8+T cells networks, respectively.

**FIGURE 4:**
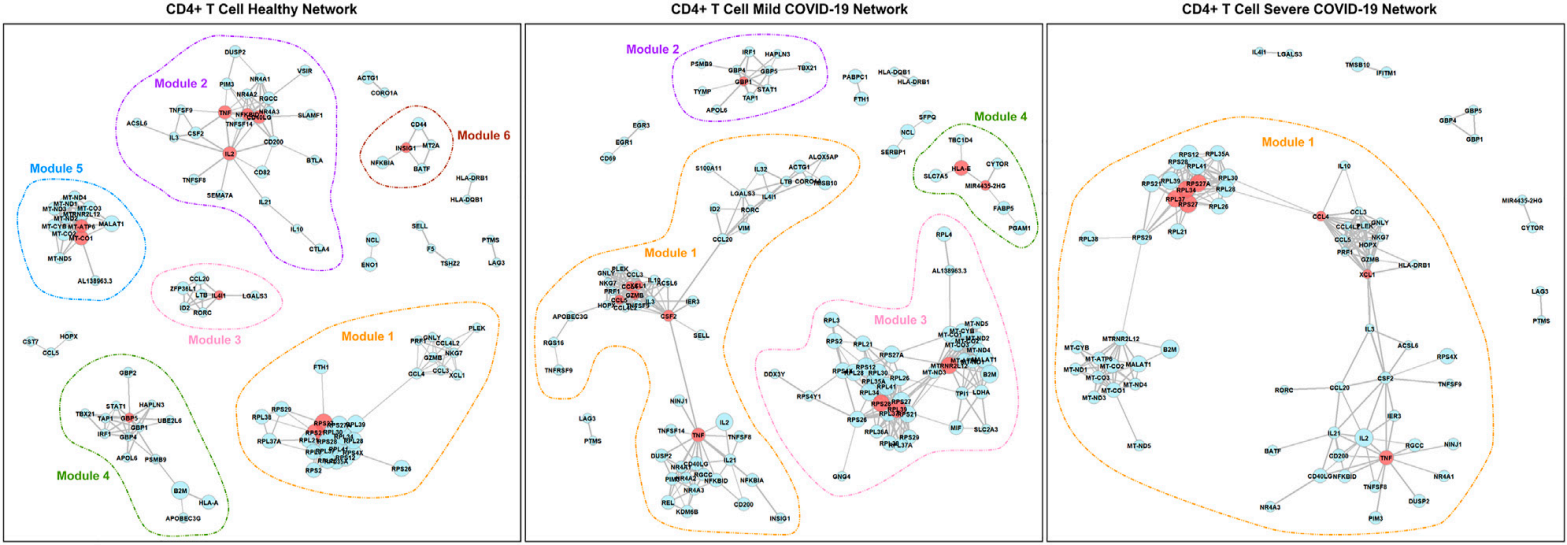
PIDC networks for CD4^+^ T cells. Node size and edge width represent the average expression level of the gene and the confidence of connectivity between a pair of genes in the network, separately. Red nodes are hub genes in the corresponding modules.

**FIGURE 5:**
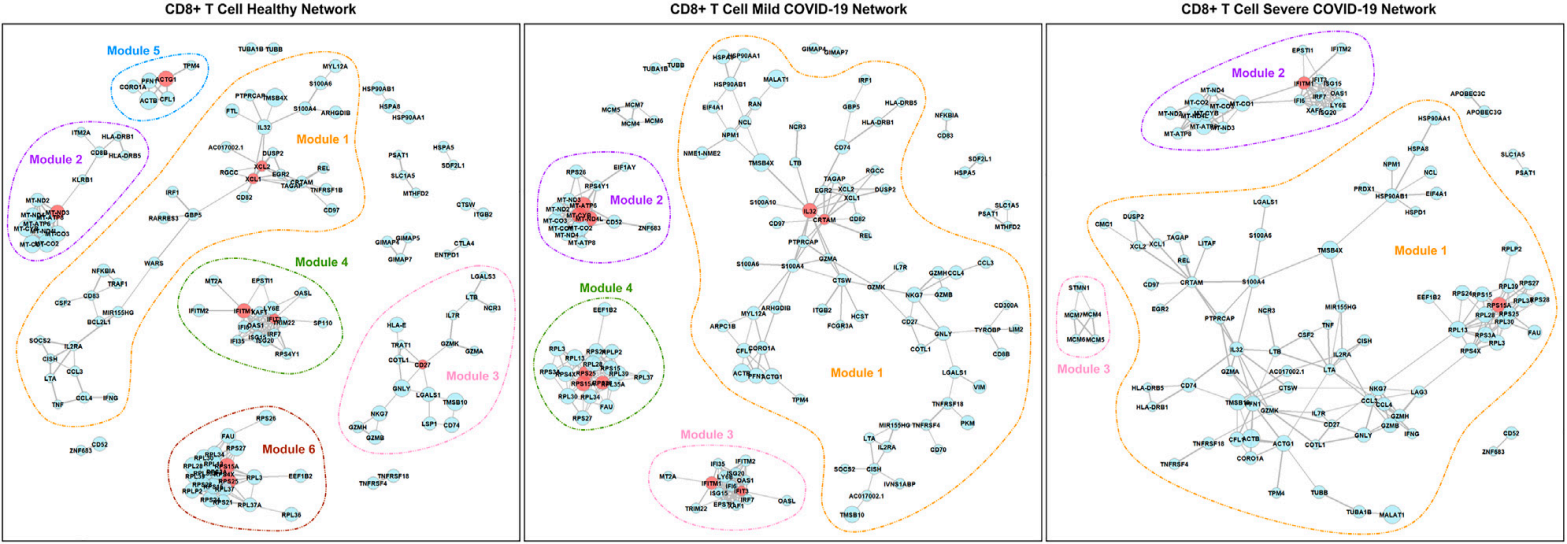
PIDC networks for CD8^+^ T cells. Node size and edge width represent the average expression level of the gene and the confidence of connectivity between a pair of genes in the network, separately. Red nodes are hub genes in the corresponding modules.

Figure 4 and Figure 5 show the networks in different disease states for CD4^+^ T cells and CD8^+^ T cells, respectively. In both cell types, we can see that the number of modules decreases in the order of healthy, mild COVID-19, and severe COVID-19. In other words, the connectivity of genes increases with the progression of the disease. This phenomenon has been noticed in many previous studies as well (Feldman et al., 2008; Gill et al., 2010). As seen in Figure 4, there is remarkable heterogeneity of the connectivity across the three networks of CD4^+^ T cells. Module 1 of the healthy network has been divided into two parts in the mild network. One part connects module 2 of the healthy network to form module 1 of the mild network, and another part connects module 5 of the healthy network to form module 3 of the mild network. And finally, all these modules link together to be a large module in the severe network. In contrast, the networks of CD8^+^ T cells seem to be more stable. This property suggests that the functions encoded by genes from CD4^+^ T cells are more varied in different disease states than CD8^+^ T cells.

According to Table 2, for CD4^+^ T cells, there are 11 hub genes in the healthy network, 12 hub genes in the mild COVID-19 network, and 7 hub genes in the severe COVID-19 network. In contrast, networks of CD8^+^ T cells contain fewer hub genes. There are 9, 10, and 2 hub genes in the healthy, mild COVID-19, and severe COVID-19 networks of CD8^+^ T cells, separately. Exploring the biological mechanism behind the connectivities of these hub genes would be helpful to understand the infection etiology.

**TABLE 2:** Hub genes in each module of each network.

Cell Type	Network	Module	Total number of genes	Hub gene
CD4^+^ T Cells	Healthy	Module 1	31	RPS21^a^, RPS27^a^
		Module 2	25	CD40LG, IL2, NFKBID, TNF
		Module 3	7	IL4I1^a^
		Module 4	15	GBP5^a^
		Module 5	13	MT-CO1^a^, MT-ATP6
		Module 6	5	INSIG1^a^
	Mild COVID-19	Module 1	53	CCL4^ac^, CCL5^ac^, GZMB^a^, TNF^c^, CSF2^c^, XCL1^a^
		Module 2	11	GBP1
		Module 3	44	RPS28^ac^, MTRNR2L12^ac^, RPL39^ac^
		Module 4	7	HLA-E^ac^, MIR4435-2HG^ac^
	Severe COVID-19	Module 1	63	RPL34^c^, CCL4^bc^, RPL37, RPS27, RPS27A^c^, TNF^c^, XCL1^b^
CD8^+^ T Cells	Healthy	Module 1	38	XCL1^ab^, XCL2^ab^
		Module 2	15	MT-ND3^ab^
		Module 3	18	CD27^ab^
		Module 4	17	IFIT3^ab^, IFITM1^d^
		Module 5	6	ACTG1
		Module 6	23	RPS25, RPS15A
	Mild COVID-19	Module 1	74	CRTAM^a^, IL32
		Module 2	15	MT-CYB^ac^, MT-ND4L^c^, MT-ATP6^c^
		Module 3	15	IFIT3^a^, IFITM1^d^
		Module 4	19	RPS25, RPS28^c^, RPS15A
	Severe COVID-19	Module 1	79	RPS15A
		Module 2	21	IFITM1^d^
		Module 3	5	-

a Significant differential connection between the healthy network and the mild COVID-19 network.

b Significant differential connection between the healthy network and the severe COVID-19 network.

c Significant differential connection between the mild COVID-19 network and the severe COVID-19 network.

d Significant differential connection in any pairwise comparison of three networks.

For CD4^+^ T cells, it is worth noting that *TNF* is a hub gene in all three networksand has the most connectivity in the corresponding module. However, its connectivities are only significantly different between the mild and the severe COVID-19 network. Its average expression level in the severe group is significantly higher than in the mild group. According to the Entrez Gene database (Maglott et al., 2010), *TNF* encodes a multifunctional proinflammatory cytokine related to multiple biological processes and diseases. Schett et al. (2020) discovered that targeting *TNF* is crucial for the inflammatory response instead of viral clearance in the cytokine pathogenesis of COVID-19. *CCL4* and *XCL1* are the hub genes in both the mild and severe networks. *CCL4* is significantly differentially connected between any pairwise comparisons among the three networks. It is also the DE gene across three disease states. On the other hand, the expression levels and connectivities of *XCL1* are both significantly different in comparing the healthy group and the COVID-19 group (either mild or severe). Actually, *CCL4* and *XCL1* are both related to chemokines and inflammatory responses (Maglott et al., 2010). Meckiff et al. (2020) also suggested that *CCL4* and *XCL1* are likely to play an essential role in the pathogenesis of COVID-19.

**FIGURE 6:**
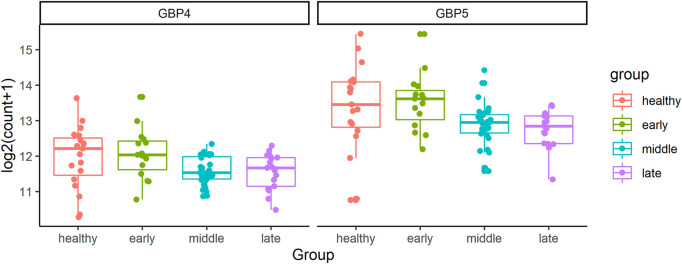
The changes of *GBP4* and *GBP5* in gene expression over time from the bulk RNA-seq data. The FDR adjusted p -values of the overall difference across three stages (using R package DESeq2) for *GBP4* and *GBP5* are 0.00156 and 0.00111, separately.

In addition, *GBP1*, *GBP4*, and *GBP5* always connect with each other in all three networks of CD4^+^ T cells. However, the number of genes associated with them decreases as the disease gets worse as shown in Figure 4. They are all responsible for encoding guanylate binding protein (Stelzer et al., 2016). Among these three genes, *GBP4* and *GBP5* are DE genes in the longitudinal bulk RNA-seq data (McClain et al., 2021). Figure 6 shows the changes of them in gene expression over time from the corresponding dataset. These two genes have higher expression levels for healthy donors and patients in the early stage of COVID-19 and show a significantly decreasing trend as time goes by (especially a significant drop at the middle stage). Interestingly, their expression levels in the healthy group are significantly lower than the disease group according to the CD4^+^ T cells data at the single-cell level. Since the longitudinal blood sample data in Figure 6 is at the bulk level, other elements different from CD4^+^ T cells may exist to make the expression level for disease patients lower than healthy donors. In particular, the expression levels of *GBP5* from CD8^+^ T cells in the disease group are significantly lower than in the healthy group.

For CD8^+^ T cells, we found that although *IFITM1* and *RPS15A* are hub genes in all three networks, they have very different properties. The connectivity of *IFITM1* is significantly different in any pairwise comparison of the three networks, while the connectivity of *RPS15A* holds the same level in the three networks. Moreover, *IFITM1* is down-regulated in the COVID-19 group (either mild or severe) compared with the healthy group, while *RPS15A* is down-regulated in the severe group compared with the healthy group. Based on the GeneCards database (Stelzer et al., 2016), *IFITM1* is highly associated with several viral diseases and can inhibit multiple infection with other enveloped viruses including coronavirus. Shi et al. (2021) identified that *IFITM1* is one of the restrictors of SARS-CoV-2 infection of cells. Although *RPS15A* is also a hub gene in all three networks of CD8^+^ T cells, it is just a ribosomal protein-coding gene that commonly exists in samples (Stelzer et al., 2016).

### 3.4 Functional Annotation

We performed functional annotation for the genes in each module of the networks with *Homo Sapiens* background using the R package clusterProfiler (Yu et al., 2012). An annotation with an FDR corrected p -value less than 0.05 was viewed as a significant annotation. We picked up the top 5 significant terms of the Gene Ontology (GO) biological process and the KEGG pathway for each module and summarized them in Tables 3, 4. The detailed dotplots for different modules in CD4^+^ and CD8^+^ T cell networks can be found in Supplementary Figures S2–S5. It is worth noting that most of the genes in module 5 of the healthy network of CD4^+^ T cells or module 2 of the healthy and mild networks of CD8^+^ T cells failed to be mapped in both the GO biological process and the KEGG pathway databases. Many of them are mitochondrial genes related to oxidative phosphorylation and translation (Errichiello et al., 2015). We also did the functional annotation for the 36 shared DE genes (Figure 2B) from the comparison of the mild and the healthy groups across CD4^+^ T cells, CD8^+^ T cells, and B cells. The results show that those genes are highly related to the viral transcription and translational initiation processes, and the COVID-19 pathway (Supplementary Figure S6).

**TABLE 3:** The main related GO biological process of each module of each network.

Cell Type	Network	Module	Number of genes	Main related biological process^a^
CD4^+^ T Cells	Healthy	Module 1	31	Viral transcription; Translational initiation
		Module 2	25	Cell-cell adhesion; Regulation of T cell and leukocyte activation; T cell proliferation
		Module 3	7	Regulation of fat cell, B cell, and myeloid cell differentiation
		Module 4	15	Response to IFN- γ ; Antigen processing and presentation
		Module 6	5	Mediation of IFN- γ ; Maintenance of protein location
	Mild COVID-19	Module 1	53	Leukocyte cell-cell adhesion; Cellular response to tumor necrosis factor; T cell proliferation
		Module 2	11	Response to IFN- γ ; Immune response
		Module 3	44	Viral transcription; Translation initiation
		Module 4	7	IL-4 production; Immune response
	Severe COVID-19	Module 1	63	Viral transcription; Translation initiation
CD8^+^ T Cells	Healthy	Module 1	38	Response to tumor necrosis factor and IFN- γ ; Regulation of cytokine production and inflammatory response
		Module 3	18	Regulation of leukocyte cell-cell adhesion and T cell activation; Cell killing; Lymphocyte apoptotic process
		Module 4	17	Response to type I IFN and virus; Negative regulation of viral genome replication
		Module 5	6	Actin filament and synapse organization
		Module 6	23	Viral transcription; Translational initiation
	Mild COVID-19	Module 1	74	Response to IFN- γ ; T cell activation; Leukocyte cell-cell adhesion
		Module 3	15	Response to type I IFN and virus; Negative regulation of viral genome replication
		Module 4	19	Viral transcription; Translational initiation
	Severe COVID-19	Module 1	79	Translational initiation; Viral transcription; Cell killing
		Module 2	21	Response to type I IFN and virus
		Module 3	5	DNA replication

a IFN: interferon; IL: interleukin.

**TABLE 4:** The main KEGG pathway of each module of each network.

Cell Type	Network	Module	Number of genes	Main KEGG pathway^a^
CD4^+^ T Cells	Healthy	Module 1	31	COVID-19
		Module 2	25	Cytokine-cytokine receptor interaction
		Module 3	7	Rheumatoid arthritis
		Module 4	15	NOD-like receptor signaling pathway
		Module 6	5	PD-L1 expression and PD-1 checkpoint pathway in cancer
	Mild COVID-19	Module 1	53	Cytokine-cytokine receptor interaction
		Module 2	11	NOD-like receptor signaling pathway
		Module 3	44	COVID-19
		Module 4	7	Central carbon metabolism in cancer
	Severe COVID-19	Module 1	63	COVID-19; Cytokine-cytokine receptor interaction
CD8^+^ T Cells	Healthy	Module 1	38	Cytokine-cytokine receptor interaction
		Module 3	18	Natural killer cell mediated cytotoxicity
		Module 4	17	COVID-19; EBV infection
		Module 5	6	Regulation of actin cytoskeleton
		Module 6	23	COVID-19
	Mild COVID-19	Module 1	74	Cytokine-cytokine receptor interaction
		Module 3	15	EBV infection
		Module 4	19	COVID-19
	Severe COVID-19	Module 1	79	COVID-19; Cytokine-cytokine receptor interaction
		Module 2	21	EBV infection
		Module 3	5	DNA replication; Cell cycle

a NOD: Nucleotide-binding oligomerization domain; PD-L1: Programmed death ligand 1; PD-1: Programmed death 1; EBV: Epstein–Barr virus.

#### 3.4.1 CD4^+^ T Cells

As we can see, functions among modules overlap with each other in the networks of CD4^+^ T cells. As seen in Tables 3 and 4, genes in module 1 of the healthy network and module 3 of the mild network can express viral transcription and some translational activities. They are also included in the COVID-19 pathway. Module 2 of the healthy network shows a crucial cluster for cellular activation and is associated with the cytokine-cytokine receptor interaction pathway. In contrast, these functions are partially displayed by genes in module 1 of the mild network only. Genes in module 4 of the healthy network and module 2 of the mild network contribute to the functions that can provide immune responses, specifically responding to IFN-γ. These genes are contained in the NOD-like receptor signaling pathway as well. The functions in module 6 of the healthy network and module 4 of the mild COVID-19 network are less critical due to the fewer genes included in these modules, but they are associated with some pathways in cancer. Since the only module in the severe network is the combination of modules from the healthy or mild network, it contains the most functions expressed in the other two networks.

Furthermore, the connectivity of genes in module 4 of the healthy network from CD4^+^ T cells gradually decreases with the progression of COVID-19 (Figure 4). Those genes are associated with several immune responses, as we discussed above. In other words, the strength of collected immune activities of these genes from CD4^+^ T cells may be lower and lower with the development of COVID-19.

#### 3.4.2 CD8^+^ T Cells

Functions among modules in the networks of CD8^+^ T cells seem to be more dispersed than CD4^+^ T cells. As shown in Table 3, genes in module 1 of the healthy network play an important role in response to tumor necrosis factor and IFN-γ. Module 3 of the healthy network mainly focuses on regulating leukocyte cell-cell adhesion and T cell activation. Cell killing is also a crucial function in module 3 and has never been expressed in CD4^+^ T cells. As for other modules of the healthy network, module 4 is highly associated with cellular defense response, module 5 is related to several fiber organizations, and module 6 tends to viral transcription. The similarity of functions among the healthy, mild, and severe networks supports our findings from the network structure. Module 3 of the severe network, a new module developed with the worsening of the disease, contributes to DNA replication activities. In addition, based on Table 4, modules 4 and 6 of the healthy network, module 4 of the mild network, and module 1 of the severe network focus on the COVID-19 pathway. Module 3 of the severe network is associated with the DNA replication pathway and the cell cycle pathway.

Because module 3 of the severe network based on CD8^+^ T cells gradually forms with the development of the disease (Figure 5), it is valuable to find a possible explanation. Several studies have reported that the SARS-CoV-2 infection will cause DNA damage (Meyer et al., 2021; Victor et al., 2021). Therefore, it is no surprise that the DNA replication and reparation functions are enhanced from the CD8^+^ T cells for COVID-19 patients.

## 4 Discussion

Although researchers have placed a lot of attention on antibody-based immunity (Cañete and Vinuesa, 2020), it will be helpful for future vaccine designs, resulting in long-term disease protection, if we know more details of the mechanism of T cell responses to SARS-CoV-2. The coronavirus-specific T cells need to be considered in vaccine strategies because it plays a critical role in controlling the development of COVID-19 and eliminating the infected virus (Tay et al., 2020).

In this study, we have applied several analytical approaches to explore the mechanism of gene expression based on CD4^+^ T cells and CD8^+^ T cells related to COVID-19. We first used MAST (Finak et al., 2015) to detect the DE genes in pairwise comparisons of the healthy, mild, and severe groups. We found that the number of DE genes between the healthy and mild groups is more than between the mild and severe groups. It indicates that the genes are expressed much differently between healthy individuals and COVID-19 groups of patients irrespective of their mild or severe disease status. In particular, we found that the average absolute value of $log_{2}FC$ across the corresponding DE genes for the severe vs mild comparison is significantly smaller than the average value for the disease vs healthy comparison. This general trend of the changes is similar to a previous study (Su et al., 2020), which found a sharper difference of immune cells between mild and moderate cases than between moderate and severe cases. The results of MLP models also validate this finding in terms of classification of samples. It indicates that the difference between the disease and healthy groups is more apparent than between severe and mild states.

After detecting the DE genes, we built networks of those DE genes specific to the sample status, such as healthy, mild, and severe networks, using the PIDC algorithm (Chan et al., 2017). We identified the topologies of the networks, such as modules for each network, hub genes for each module, and evaluated the difference in connectivity of each gene in three different networks. Based on the gene co-expression networks, we found that the connectivity of genes increases in both CD4^+^ and CD8^+^ T cells as the disease symptoms worsen. This relationship between the connectivity of genes and the severity of the disease has been noticed in many previous studies as well (Feldman et al., 2008; Gill et al., 2010).

In general, we found genes *TNF*, *CCL4*, and *XCL1* can be highly identified with SARS-CoV-2 based on CD4^+^ T cells, while *IFITM1* plays an essential role in fighting with SARS-CoV-2 from CD8^+^ T cells. On the other hand, the gene co-expression networks and the corresponding functional annotations let us know some crucial clusters of genes related to COVID-19. For CD4^+^ T cells, genes in module 1 of the healthy network, module 3 of the mild network, and module 1 of the severe network are worth noticing. For CD8^+^ T cells, genes in modules 4 and 6 of the healthy network, module 4 of the mild network, and module 1 of the severe network are nonnegligible. The overall results of this study might improve the insight of gene expression mechanisms associated with T cells and expand our understanding of the molecular mechanism of COVID-19.

As time progresses, SARS-CoV-2 virus evolves with multiple variants, such as Alpha, Beta, Delta, and Omicron, have spread worldwide. Specifically, the Delta and Omicron variants have extremely high infectivity, but the Omicron variant is less likely to cause severe disease (Campbell et al., 2021; Haque and Pant, 2022). Although the protective efficacy of existing vaccines against the variants has a certain degree of protective efficacy, people still need to develop vaccines which can protect against multiple strains of the virus and the effect lasts for a longer period (Bian et al., 2021; Nemet et al., 2021). Hence, the immunity related cells, such as CD4^+^ T cells and CD8^+^ T cells, are extremely important. Due to the limitation of available data, we could not identify the viral strain-specific differences for the T cell responses. Consequently, further studies of the correlation of immune cells, including T cells and B cells, and the specific variants of SARS-CoV-2 are necessary. That will also help for future vaccine development for long-term protection for all variants of the virus.

## Data Availability

Publicly available datasets were analyzed in this study. This data can be found here: GEO database repository (https://www.ncbi.nlm.nih.gov/geo). Specifically, the CD4^+^ T cells, CD8^+^ T cells, B cells, and longitudinal bulk data are available under accession numbers GSE162086, GSE153931, GSE164381, and GSE161731, respectively.
