# Supplementary material for: Unraveling T Cell Responses for Long Term Protection of SARS-CoV-2 Infection
Source: Front Genet. 2022 May 4;13:871164. doi: 10.3389/fgene.2022.871164 (PMC9114762; doi:10.3389/fgene.2022.871164)
Supplement: Supplementary file 1 [file DataSheet1.zip › Supplementary_Material.docx]

Supplementary Material

# Supplementary Data

The following files contain the lists of differentially expressed (DE) genes for each comparison.

**DEGenes_CD4+TCell.xlsx:** DE genes of pairwise comparisons among the healthy, mild, and severe groups from CD4+ T cells

**DEGenes_CD8+TCell.xlsx:** DE genes of pairwise comparisons among the healthy, mild, and severe groups from CD8+ T cells

**DEGenes_BCell.xlsx:** DE genes of the comparison between the healthy and mild groups from B cells

# Supplementary Figures and Tables

## Supplementary Figures


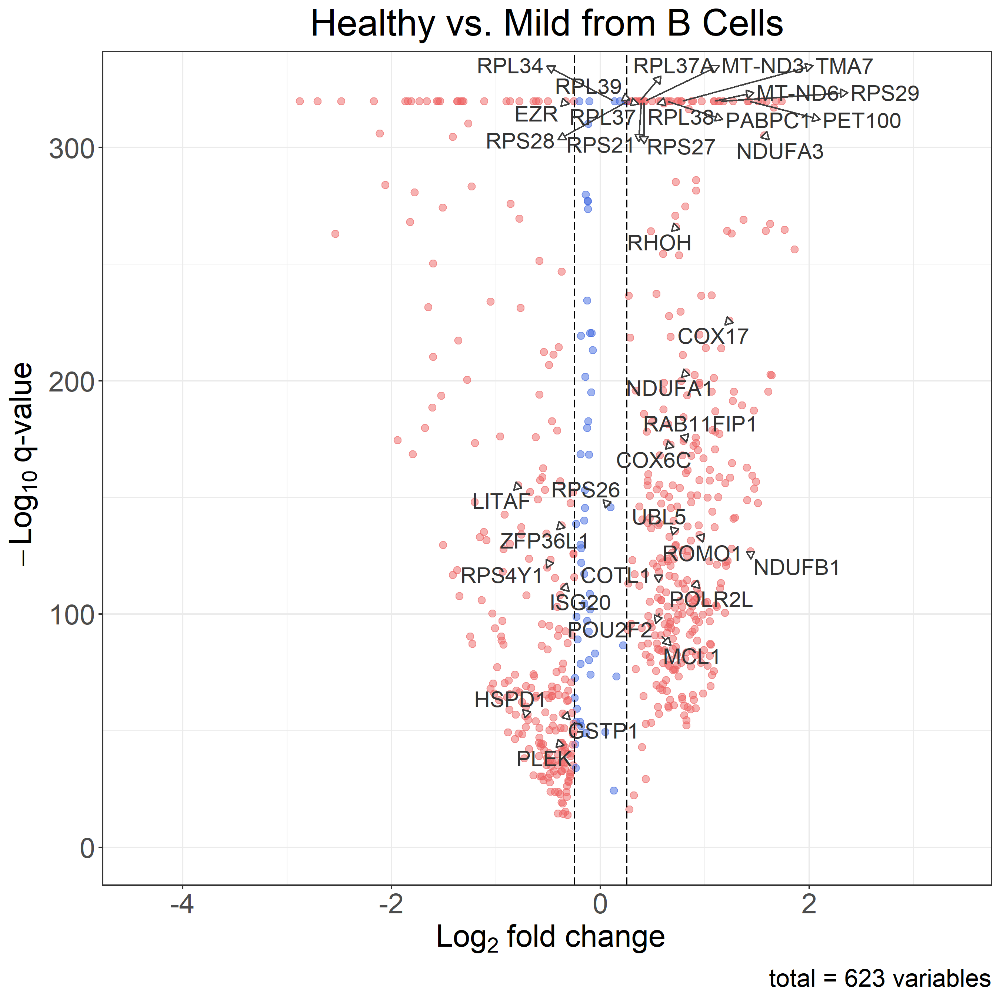


**Supplementary Figure S1**. The volcano plot for DE genes of mild vs. healthy based on B cells. The threshold of the absolute $\text{log}_{\text{2}}FC$ is 0.25. The shared DE genes of healthy vs. mild across CD4+ T cells, CD8+ T cells, and B cells are labeled in the plot.


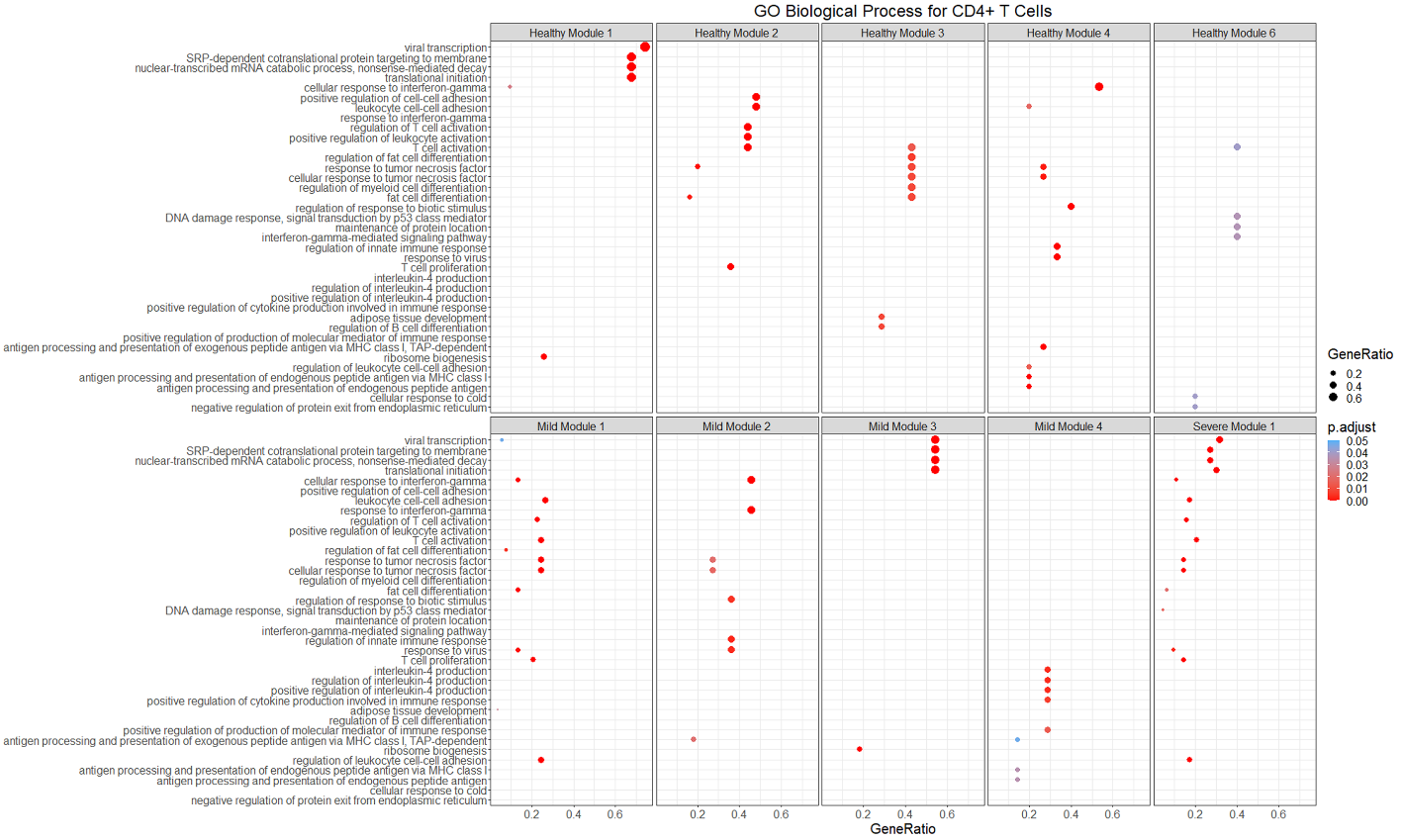


**Supplementary Figure S2**. Dotplots of the top 5 significant terms from the GO biological process for different modules in three CD4+ T cell networks. As long as these terms are significant in other modules, we still displayed them in the figures. Gene ratio is the ratio of the number of genes related to the specific term and the total number of genes in this module.


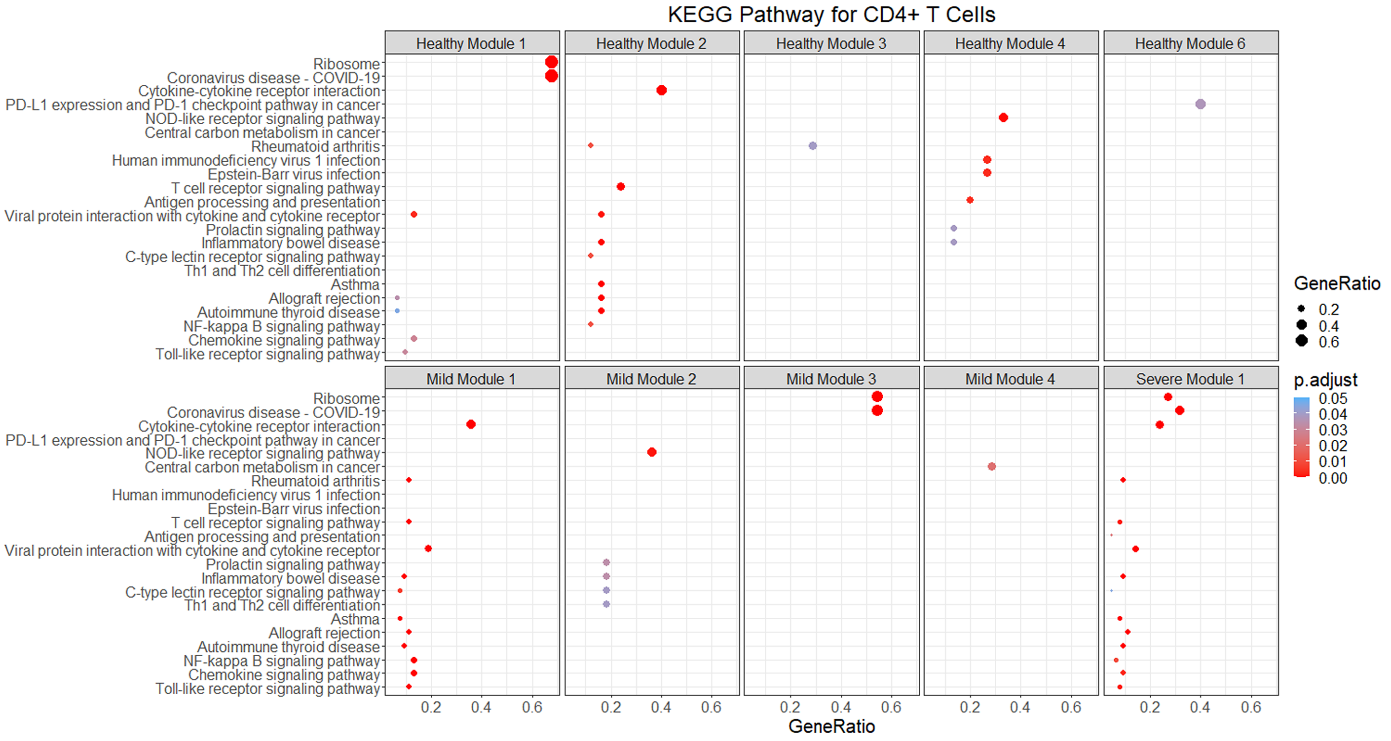


**Supplementary Figure S3**. Dotplots of the top 5 significant terms from the KEGG pathway for different modules in three CD4+ T cell networks. As long as these terms are significant in other modules, we still displayed them in the figures. Gene ratio is the ratio of the number of genes related to the specific term and the total number of genes in this module.


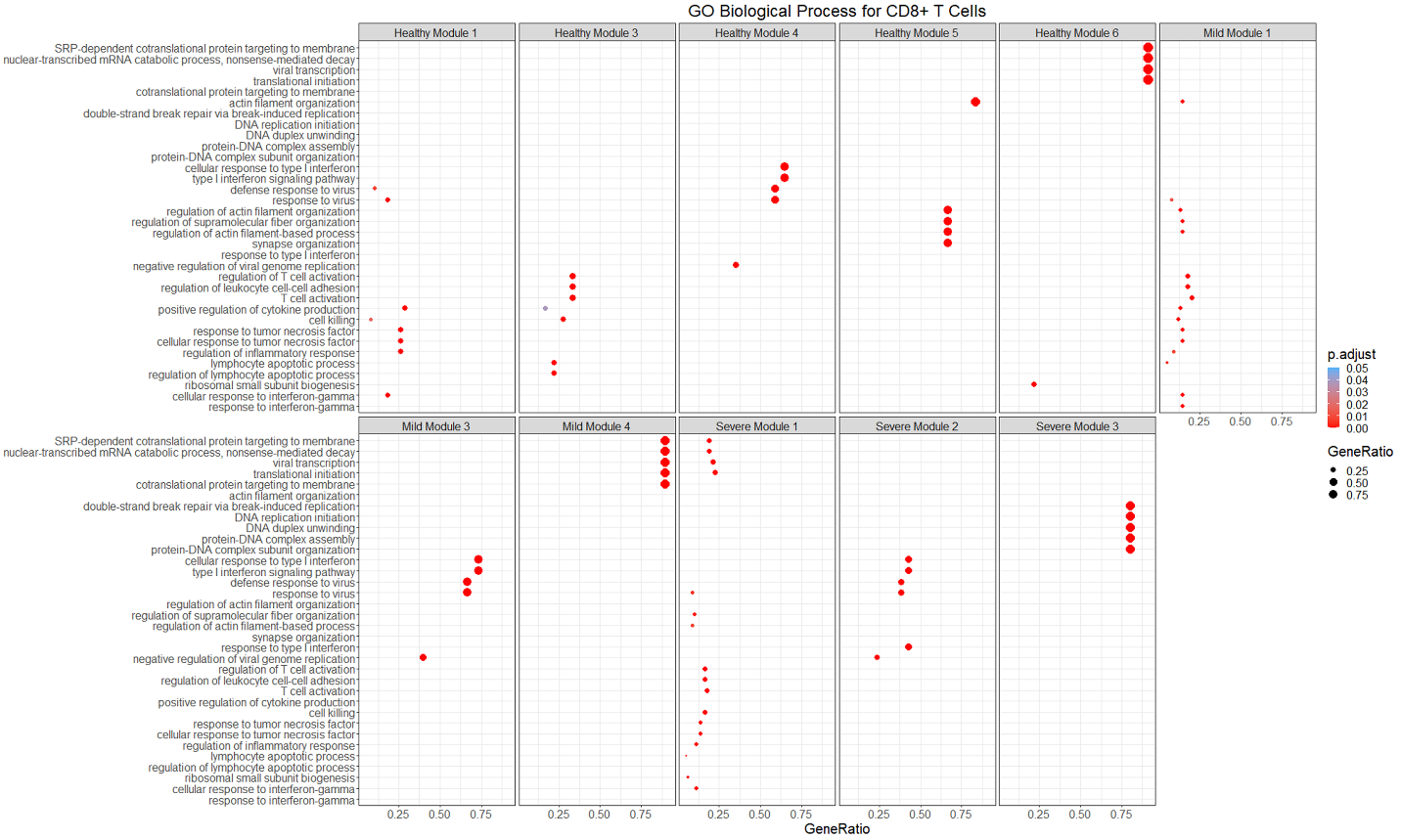


**Supplementary Figure S4**. Dotplots of the top 5 significant terms from the GO biological process for different modules in three CD8+ T cell networks. As long as these terms are significant in other modules, we still displayed them in the figures. Gene ratio is the ratio of the number of genes related to the specific term and the total number of genes in this module.


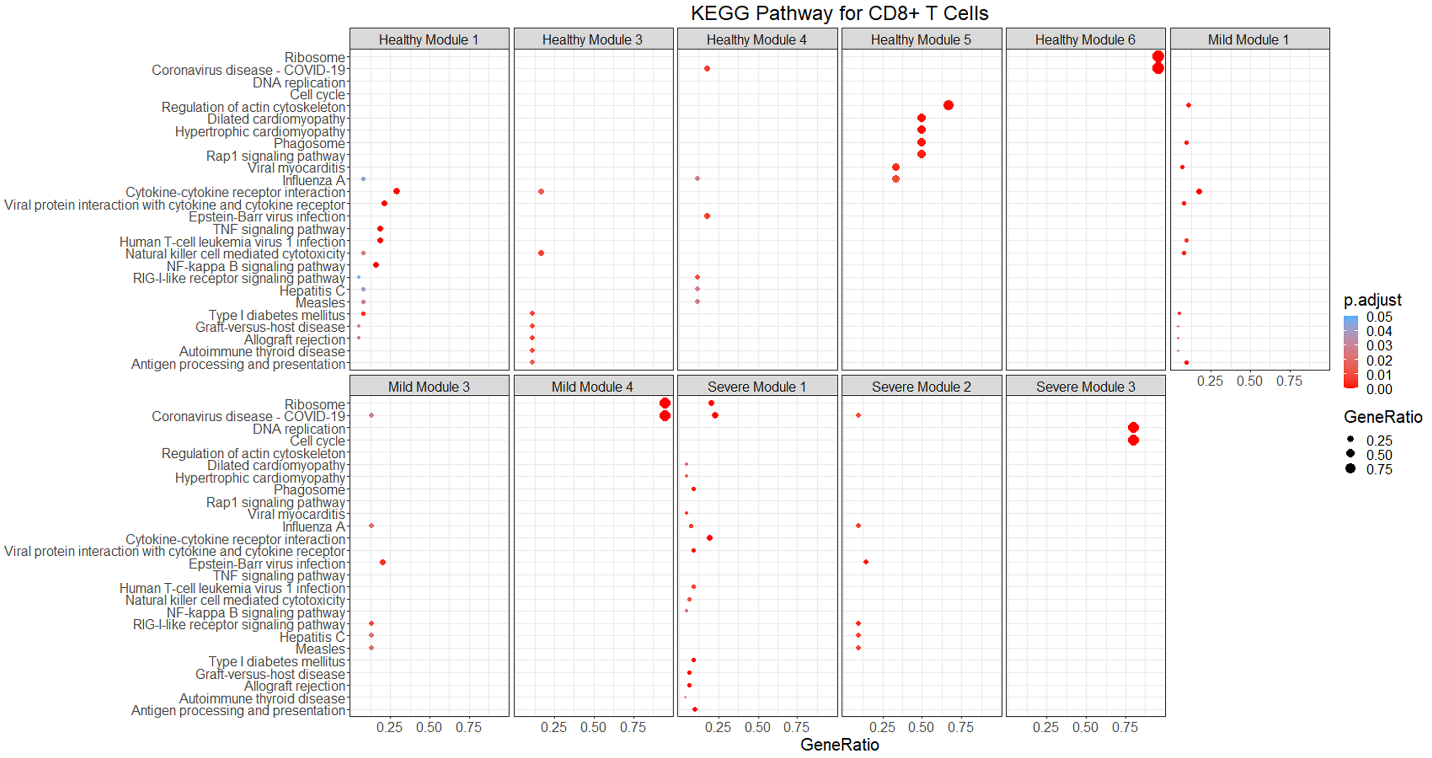


**Supplementary Figure S5**. Dotplots of the top 5 significant terms from the KEGG pathway for different modules in three CD8+ T cell networks. As long as these terms are significant in other modules, we still displayed them in the figures. Gene ratio is the ratio of the number of genes related to the specific term and the total number of genes in this module.


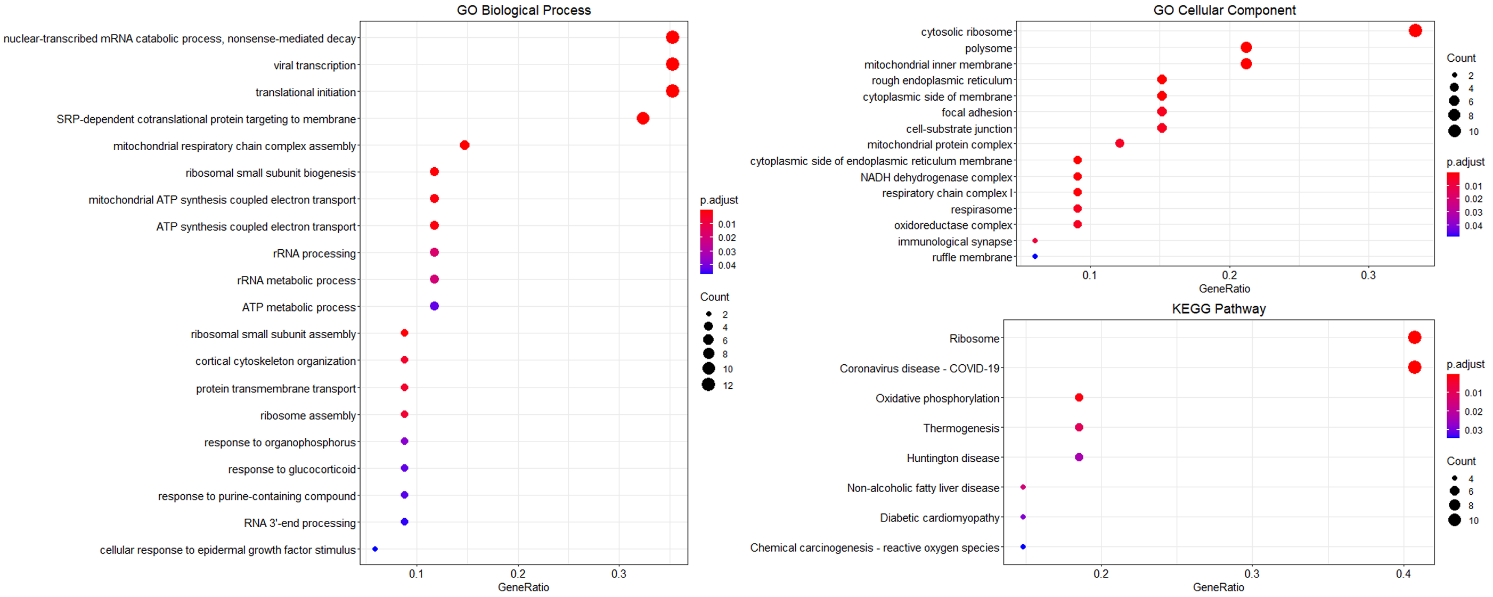


**Supplementary Figure S6.** The functional annotation results for the 36 shared DE genes from the comparison of the mild and the healthy groups across CD4+ T cells, CD8+ T cells, and B cells.

## Supplementary Tables

**Supplementary Table S1.** The selected MLP models for each comparison. H, M, and S indicate the healthy group, the mild COVID-19 group, and the severe COVID-19 group, respectively.

| Cell type | Comparison | Optimizer | If using weight |
| --- | --- | --- | --- |
| CD4+ T cells | M vs H | adam | No |
|  | S vs H | rmsprop | No |
|  | S vs M | adam | No |
| CD8+ T cells | M vs H | rmsprop | No |
|  | S vs H | rmsprop | No |
|  | S vs M | adam | No |
| B cells | M vs H | adam | No |

**Supplementary Table S2.** The classification results of the mild and healthy groups from CD4+ T cells. H and M indicate the healthy group and the mild COVID-19 group, respectively.

| ID | Group | # of cells | # of cells correctly predicted | Accuracy |
| --- | --- | --- | --- | --- |
| J15891 | H | 754 | 704 | 0.9337 |
| J15892 | H | 1113 | 962 | 0.8643 |
| J15899 | H | 376 | 359 | 0.9548 |
| J15900 | H | 296 | 272 | 0.9189 |
| J21855 | H | 587 | 477 | 0.8126 |
| J21856 | H | 831 | 765 | 0.9206 |
| J09835 | M | 1220 | 1216 | 0.9967 |
| J09836 | M | 1327 | 1327 | 1.0000 |
| J10624 | M | 7696 | 7671 | 0.9968 |
| J10625 | M | 5648 | 5027 | 0.8900 |
| J11689 | M | 3059 | 2438 | 0.7970 |
| J15890 | M | 4623 | 3742 | 0.8094 |

**Supplementary Table S3.** The classification results of the severe and healthy groups from CD4+ T cells. H and S indicate the healthy group and the severe COVID-19 group, respectively.

| ID | Group | # of cells | # of cells correctly predicted | Accuracy |
| --- | --- | --- | --- | --- |
| J15891 | H | 754 | 704 | 0.9337 |
| J15892 | H | 1113 | 982 | 0.8823 |
| J15899 | H | 376 | 352 | 0.9362 |
| J15900 | H | 296 | 269 | 0.9088 |
| J21855 | H | 587 | 175 | 0.2981 |
| J21856 | H | 831 | 723 | 0.8700 |
| J10535 | S | 11585 | 11516 | 0.9940 |
| J10886 | S | 5084 | 5081 | 0.9994 |
| J10887 | S | 4151 | 4079 | 0.9827 |
| J10888 | S | 3669 | 3483 | 0.9493 |
| J14204 | S | 6909 | 6540 | 0.9466 |
| J14205 | S | 9961 | 9899 | 0.9938 |
| J15893 | S | 17915 | 17891 | 0.9987 |
| J21854 | S | 17613 | 17395 | 0.9876 |

**Supplementary Table S4.** The classification results of the severe and mild groups from CD4+ T cells. M and S indicate the mild COVID-19 group and the severe COVID-19 group, respectively.

| ID | Group | # of cells | # of cells correctly predicted | Accuracy |
| --- | --- | --- | --- | --- |
| J10535 | S | 11585 | 5377 | 0.4641 |
| J10886 | S | 5084 | 2051 | 0.4034 |
| J10887 | S | 4151 | 3240 | 0.7805 |
| J10888 | S | 3669 | 3342 | 0.9109 |
| J14204 | S | 6909 | 6610 | 0.9567 |
| J14205 | S | 9961 | 9757 | 0.9795 |
| J15893 | S | 17915 | 16782 | 0.9368 |
| J21854 | S | 17613 | 17406 | 0.9882 |
| J09835 | M | 1220 | 1045 | 0.8566 |
| J09836 | M | 1327 | 1207 | 0.9096 |
| J10624 | M | 7696 | 6592 | 0.8565 |
| J10625 | M | 5648 | 4649 | 0.8231 |
| J11689 | M | 3059 | 2503 | 0.8182 |
| J15890 | M | 4623 | 512 | 0.1108 |

**Supplementary Table S5.** The classification results of the mild and healthy groups from CD8+ T cells. H and M indicate the healthy group and the mild COVID-19 group, respectively.

| ID | Group | # of cells | # of cells correctly predicted | Accuracy |
| --- | --- | --- | --- | --- |
| SDBB082 | H | 1659 | 1590 | 0.9584 |
| SDBB088 | H | 1532 | 1505 | 0.9824 |
| SDBB051 | H | 422 | 408 | 0.9668 |
| SDBB079 | H | 1015 | 945 | 0.9310 |
| P25 | M | 2798 | 2740 | 0.9793 |
| P26 | M | 76 | 69 | 0.9079 |
| P29 | M | 485 | 481 | 0.9918 |
| P44 | M | 412 | 412 | 1.0000 |
| P37 | M | 75 | 75 | 1.0000 |
| P45 | M | 171 | 167 | 0.9766 |
| P31 | M | 20 | 20 | 1.0000 |
| P40 | M | 407 | 389 | 0.9558 |
| P22 | M | 4735 | 4713 | 0.9954 |
| P32 | M | 763 | 756 | 0.9908 |
| P47 | M | 647 | 647 | 1.0000 |
| P66 | M | 1015 | 1013 | 0.9980 |
| P57 | M | 597 | 595 | 0.9966 |
| P64 | M | 845 | 845 | 1.0000 |
| P61 | M | 62 | 60 | 0.9677 |

**Supplementary Table S6.** The classification results of the severe and healthy groups from CD8+ T cells. H and S indicate the healthy group and the severe COVID-19 group, respectively.

| ID | Group | # of cells | # of cells correctly predicted | Accuracy |
| --- | --- | --- | --- | --- |
| SDBB082 | H | 1659 | 1399 | 0.8433 |
| SDBB088 | H | 1532 | 1263 | 0.8244 |
| SDBB051 | H | 422 | 389 | 0.9218 |
| SDBB079 | H | 1015 | 868 | 0.8552 |
| P10 | S | 3146 | 3143 | 0.9990 |
| P12 | S | 3739 | 3739 | 1.0000 |
| P07 | S | 3037 | 3037 | 1.0000 |
| P05 | S | 2285 | 2282 | 0.9987 |
| P06 | S | 3630 | 3630 | 1.0000 |
| P08 | S | 9806 | 9802 | 0.9996 |
| P24 | S | 7076 | 7067 | 0.9987 |
| P09 | S | 1902 | 1902 | 1.0000 |
| P03 | S | 3497 | 3497 | 1.0000 |
| P04 | S | 245 | 243 | 0.9918 |
| P01 | S | 557 | 557 | 1.0000 |
| P20 | S | 1855 | 1854 | 0.9995 |
| P15 | S | 618 | 615 | 0.9951 |
| P16 | S | 2465 | 2456 | 0.9963 |
| P17 | S | 867 | 851 | 0.9815 |
| P18 | S | 263 | 260 | 0.9886 |
| P19 | S | 369 | 361 | 0.9783 |
| P43 | S | 2987 | 2921 | 0.9779 |
| P46 | S | 617 | 585 | 0.9481 |
| P49 | S | 5257 | 4683 | 0.8908 |
| P42 | S | 946 | 937 | 0.9905 |
| P27 | S | 5 | 5 | 1.0000 |

**Supplementary Table S7.** The classification results of the severe and mild groups from CD8+ T cells. M and S indicate the mild COVID-19 group and the severe COVID-19 group, respectively.

| ID | Group | # of cells | # of cells correctly predicted | Accuracy |
| --- | --- | --- | --- | --- |
| P10 | S | 3146 | 2469 | 0.7848 |
| P12 | S | 3739 | 2289 | 0.6122 |
| P07 | S | 3037 | 2474 | 0.8146 |
| P05 | S | 2285 | 1393 | 0.6096 |
| P06 | S | 3630 | 3116 | 0.8584 |
| P24 | S | 7076 | 6500 | 0.9186 |
| P08 | S | 9806 | 9661 | 0.9852 |
| P09 | S | 1902 | 1784 | 0.9380 |
| P03 | S | 3497 | 3207 | 0.9171 |
| P01 | S | 557 | 495 | 0.8887 |
| P04 | S | 245 | 233 | 0.9510 |
| P20 | S | 1855 | 1376 | 0.7418 |
| P15 | S | 618 | 550 | 0.8900 |
| P16 | S | 2465 | 1854 | 0.7521 |
| P17 | S | 867 | 307 | 0.3541 |
| P18 | S | 263 | 167 | 0.6350 |
| P19 | S | 369 | 253 | 0.6856 |
| P49 | S | 5257 | 4748 | 0.9032 |
| P43 | S | 2987 | 2645 | 0.8855 |
| P46 | S | 617 | 573 | 0.9287 |
| P42 | S | 946 | 909 | 0.9609 |
| P27 | S | 5 | 4 | 0.8000 |
| P25 | M | 2798 | 1400 | 0.5004 |
| P26 | M | 76 | 27 | 0.3553 |
| P29 | M | 485 | 321 | 0.6619 |
| P44 | M | 412 | 287 | 0.6966 |
| P37 | M | 75 | 40 | 0.5333 |
| P31 | M | 20 | 14 | 0.7000 |
| P40 | M | 407 | 152 | 0.3735 |
| P45 | M | 171 | 63 | 0.3684 |
| P22 | M | 4735 | 461 | 0.0974 |
| P32 | M | 763 | 259 | 0.3394 |
| P66 | M | 1015 | 540 | 0.5320 |
| P64 | M | 845 | 461 | 0.5456 |
| P57 | M | 597 | 321 | 0.5377 |
| P47 | M | 647 | 456 | 0.7048 |
| P61 | M | 62 | 49 | 0.7903 |

**Supplementary Table S8.** The classification results of the mild and healthy groups from B cells. H and M indicate the healthy group and the mild COVID-19 group, respectively.

| ID | Group | # of cells | # of cells correctly predicted | Accuracy |
| --- | --- | --- | --- | --- |
| GSM5008746 | H | 4032 | 3754 | 0.9311 |
| GSM5008747 | H | 3554 | 3366 | 0.9471 |
| GSM5008748 | H | 7426 | 7412 | 0.9981 |
| GSM5008749 | H | 2350 | 2298 | 0.9779 |
| GSM5008750 | M | 3946 | 3881 | 0.9835 |
| GSM5008751 | M | 2443 | 2410 | 0.9865 |
| GSM5008752 | M | 2311 | 2259 | 0.9775 |
| GSM5008753 | M | 1620 | 1601 | 0.9883 |
| GSM5008754 | M | 1701 | 1677 | 0.9859 |
| GSM5008755 | M | 1191 | 1171 | 0.9832 |
| GSM5008756 | M | 4202 | 4166 | 0.9914 |
